# BDMCA: a big data management system for Chinese auditing

**DOI:** 10.7717/peerj-cs.1317

**Published:** 2023-04-13

**Authors:** Xiaoping Zhou, Bin Ge, Zeyu Xia, Weidong Xiao, Zhiya Chen

**Affiliations:** 1 Central South University, Business School, Changsha, Hunan, China; 2 National University of Defense Technology, Science and Technology on Information System Engineering Laboratory, Changsha, Hunan, China; 3 National University of Defense Technology, School of Computer, Changsha, Hunan, China

**Keywords:** Chinese audits, Big data, X-SQL

## Abstract

The advent of big data technologies makes a profound impact on various facets of our lives, which also presents an opportunity for Chinese audits. However, the heterogeneity of multi-source audit data, the intricacy of converting Chinese into SQL, and the inefficiency of data processing methods present significant obstacles to the growth of Chinese audits. In this article, we proposed BDMCA, a big data management system designed for Chinese audits. We developed a hybrid management architecture for handling Chinese audit big data, that can alleviate the heterogeneity of multi-mode data. Moreover, we defined an R-HBase spatio-temporal meta-structure for auditing purposes, which exhibits almost linear response time and excellent scalability. Compared to MD-HBase, R-HBase performs 4.5× and 3× better in range query and kNN query, respectively. In addition, we leveraged the slot value filling method to generate templates and build a multi-topic presentation learning model MRo-SQL. MRo-SQL outperforms the state-of-the-art X-SQL parsing model with improvements in logical-form accuracy of up to 5.2%, and execution accuracy of up to 5.9%.

## Introduction

Prominent websites currently process thousands of petabytes (PB) data on a daily basis. This quantity of data is rapidly expanding, with an increasingly diverse range of data types being generated. Additionally, there is a growing demand for real-time processing, createing a huge gap between data processing and demand.

Big data is a conceptual framework that encompasses several distinct characteristics, including high-speed data flow, diverse data types, a low value density, and extensive data scales (Holt & Loraas, 2021). Its scope extends far beyond what traditional data analysis software can process. With the rapid advancement of information technology in modern times, the utilization of information in society has markedly improved, leading to the production of valuable knowledge and services. In comparison to traditional data warehousing applications, big data presents a more extensive array of data types, larger volumes, and more intricate query analyses (Zeyu et al., 2020). It has the capability to provide various data and analysis results in real-time, which can be applied in the decision-making process.

Computer-aided audits involve using computers to carry out activities such as economic supervision, verification, and evaluation. The primary aim of computer audits, which encompasses information system audits and computer-aided audits, is to scrutinize data and its implications (Quezada-Sarmiento, Alvarado-Camacho & Chango-Cañaveral, 2017). With the emergence of the Internet, big data, cloud computing, and other related technologies, the computer-audit discipline has evolved. One significant theory pertains to the utilization of big data technology, which involves advanced information technology, to carry out online auditing (Yoon, Hoogduin & Zhang, 2015). To this end, an audit platform is established for collecting data on each audit object, which is then processed and cleansed. By analyzing and studying these data, auditors can resolve many uncertainties that arise.

Efficient data management techniques have always been a key focus of research in the field of data engineering, with big data having a significant impact on conventional data sources, processing methods, and thinking patterns. Multi-database systems, which are primarily designed to address the issue of interoperability between homologous relational databases, deal with problems related to query processing and optimization (Özsu & Valduriez, 1999). To establish a strong connection between Hadoop and data warehouses, Xu, Kostamaa & Gao (2010) and Abouzeid et al. (2009) proposed a solution that transfers results to the appropriate database based on the users’ query requirements, thus allowing access to data of different types. Tian et al. (2015) envision that traditional data warehouses and Hadoop’s distributed file system (HDFS) will have a bright future, particularly in the domains of business and financial analysis, where integrating the two databases will become a common practice to enhance efficiency. A query module for accessing distributed heterogeneous databases was presented in Tomasic, Raschid & Valduriez (1998), thereby resolving the tightly-coupled problem between the query and the data source.

Current research on multi-dimensional indexing techniques focuses primarily on areas such as distributed indexing, temporal and spatial indexing, and cloud indexing. The study of SIMPEER (Doulkeridis et al., 2007) is centered around the calculation of metric space similarity search using peer methods. Hernandez, Rodriguez & Marin (2008) presents a meta-index mechanism that enables the creation of a global distributed index, providing the system with robust fault tolerance, but does not address the issue of load balancing. The differences in time and space partitioning are discussed and a strategy of “the older the spatial information, the coarser the partitioning” is proposed (Nah et al., 2005). A spatial information transfer algorithm is designed based on this strategy, and methods for managing effective time are also suggested to mitigate inaccuracies in data transfer. BORA (Bresenham-based Overlay for Routing and Aggregation) (Trajcevski et al., 2007) is an indexing architecture that leverages distributed technology to handle regional queries. It converts spatial mapping into a grid-like structure and uses the Bresenham algorithm to aggregate track segments found in relevant servers within the grid, providing results for regional queries.

Text2SQL is a cutting-edge semantic parsing technology that transforms natural language questions into SQL statements, significantly lowering the barrier to entry for database usage and enhancing information utilization efficiency. In the early (Green et al. 1961) pioneered the development of a natural language query interface for the BASEBALL system, which was designed to work specifically with databases. Liang, Jordan & Klein (2013) investigated how to map natural language questions to answers through latent logical representations, resulting in the creation of a new semantic representation method called DCS. In 2016, Jia & Liang (2016) posited that prior knowledge plays a crucial role in neural semantic parsing models and introduced a data reorganization framework that incorporates this type of prior knowledge into the parsing process. Fan et al. (2017) explored the application of transfer learning to neural semantic parsing technology. In recent years, pre-training techniques such as BERT (Devlin et al., 2018), ELMo (Sarzynska-Wawer et al., 2021), and GPT (Radford et al., 2018) have made tremendous strides in multiple natural language processing tasks. Hwang et al. (2019) believe that pre-training is a key factor in achieving high accuracy in NLP tasks and have examined the use of the BERT pre-trained model in the Text2SQL task, proposing three distinct variants. Guo & Gao (2019) leveraged the BERT pre-trained model to develop a straightforward table content utilization network for the Text2SQL task.

At present, the main difficulties faced by Chinese big data audits include (i) the unified management of massive multi-source heterogeneous data, (ii) fast retrieval of required information, and (iii) transformation of Chinese semantics into the corresponding SQL language. To solve these problems, we proposed BDMCA, a Big Data Management System for Chinese Audit. The main contributions of this article are:

- We innovatively designed a multi-source heterogeneous hybrid data management framework. The framework integrats multi-source, heterogeneous, multidimensional and high-level Chinese audit data into a single architecture, making unified management of heterogeneous data possible.
- We proposed a skeleton-based index construction and R-tree-based retrieval method. Considering the heterogeneous characteristics of the audit data, and the requirement for multidimensional indexing, we utilized skeleton indexes to retrieve time and space dimensions, and integrated R-Tree to address the lack of secondary indexing capabilities in HBase.
- We proposed an intelligent audit data retrieval method MRo-SQL, based on Chinese-assisted SQL statement generation. MRo-SQL involves the NL2SQL generation template using slot filling, the construction of a multi-topic representation learning model, and the design of a multi-task joint learning architecture. By designing effective encoders, column representation modules, and sub-model units, this approach shows improved generalization performance.

The remainding of this article is organized as follows. “Materials and methods” introduces technical methods and related principles used by BDMCA. “Results” evaluates the system. “Conclusion and future work” summarizes the article and outlines avenues for future research.

## Materials and Methods

### Heterogeneous hybrid data management framework

To optimize the Chinese audit big data mode, it is crucial to implement appropriate reforms and enhancements to data management in accordance with the latest developments in auditing. This section presents an effective hybrid management framework for the audit big data. The proposed management architecture is based on the mediator-wrapper management architecture and focuses on defining the mediators, particularly with respect to the segmentation, storage management, query evaluation, and execution of audit data slices. Through mediator coordination, a middleware layer is established to facilitate the successful implementation of the hybrid management.

**Figure 1:**
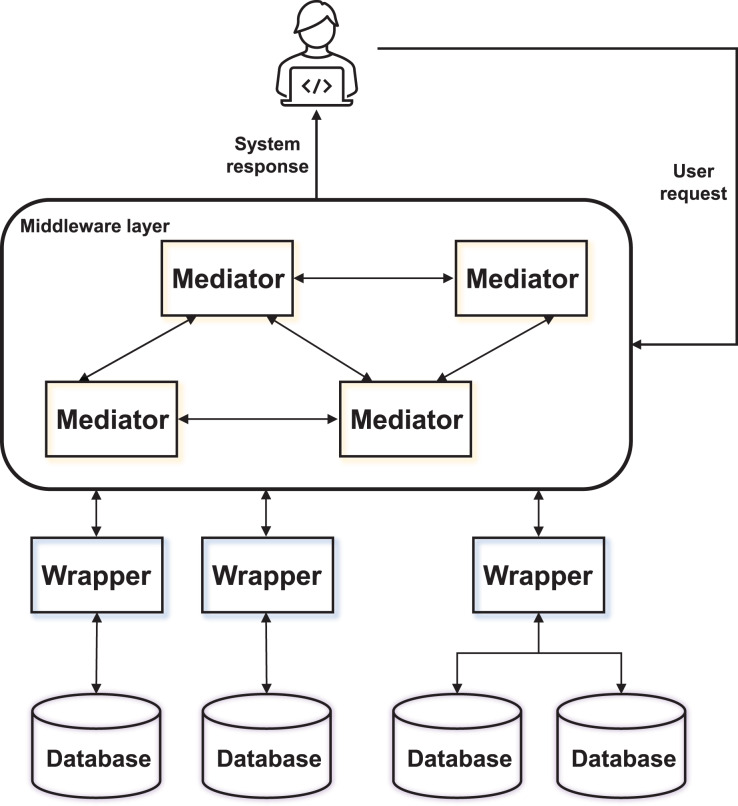
The architecture of the mediator-wrapper system.

The present system employs a mediator-wrapper management architecture to effectively manage the segmentation, storage, query evaluation, and execution of audit data slices by defining the mediators. The middleware layer is established through mediator coordination to implement a hybrid management scheme. Figure 1 depicts a representative mediator-wrapper system.

The proposed system facilitates the distribution of audit data to different wrappers and databases *via* mediators, allowing for adaptive adjustments based on the specific audit tasks and requirements. This feature significantly enhances the convenience of subsequent audit data queries and processing. To address the unique characteristics of massive and multimodal audit data, the system defines four distinct mediators: storage instructors, storage description managers, query evaluators, and runtime actuators. The storage instructor fragmentizes each audit dataset into overlapping segments and assigns storage to one of the available data management systems. The storage description manager is responsible for maintaining the directories of available storage data slices. The query evaluator processes the query request in the original language of the data source distributed by the application. Finally, the runtime executor converts the overrides into physical plans at runtime, enabling direct execution.

Within the system, audit data are stored in various data stores, and the execution of query requests is split between the mediator and the wrapper. Unlike the mediator, this architecture disperses the data across different models and selects combinations from the available systems to enhance the overall system performance.

**Figure 2:**
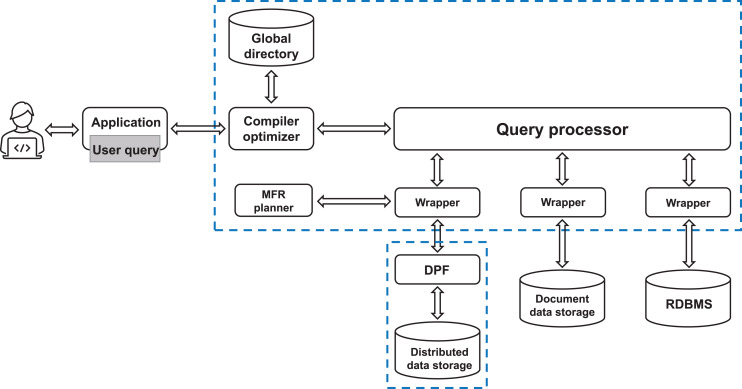
The architecture of the query engine system.

The audit process relies on the SQL database query language, and the audit system conducts data query processing. To tackle the challenge of heterogeneous data integration and query processing, the system adopts a mediator/wrapper architecture. Queries are capable of integrating data across multiple data stores, including structured (relational database), semi-structured (document database), and unstructured (distributed storage based on HDFS) data. Figure 2 illustrates the query engine system architecture.

The architecture comprises a compiler, a general query processor (mediation), three wrappers, and data storage structures (three distributed DPFs, an RDBMS, and a document database). The DPF is tasked with executing parallel data processing on distributed data stores. Within this architecture, each data source is paired with a corresponding wrapper that performs subqueries on the data store and converts the retrieved dataset into tables that match the number and type of columns requested, thus enabling the tables to be utilized by relational operators.

With the utilization of the above technological architecture, it is possible not only to improve the efficiency of data storage, querying, and processing, but also to advance the networked auditing mode quickly. In this architecture, auditing agencies can efficiently access new types of data that are highly applicable to auditing, and perform fast analysis and preprocessing of the data to accelerate the auditing process and identify auditing suspicions at the earliest possible time. As a result, the architecture proposed in this section is able to adapt to heterogeneous data dynamically, performs fast queries in the data processing procedure, and effectively reduces the auditing time.

### Skeleton-based index construction and R-tree-based retrieval

With the depth and breadth of audit expands, the data types, value ranges, and structures of data are more and more complicated. To deal with this problem, we developed a spatio-temporal meta-structure of audit data. Then, in order to make metadata audit resources more convenient for unified retrieval, we designed a *skeleton index* structure, which features a loosely coupled but efficient index linkage mechanism. Additionally, as HBase does not have inherent secondary indexing capabilities, we utilized R-Tree to address high-dimensional spatial retrieval challenges and proposed an innovative solution, R-HBase (R-Tree Hadoop Database), that employs a time-space element structure based on HBase for storing audits.

#### A spatio-temporal meta-structure of audit data

In BDMCA, the data to be audited is treated as a set. According to certain classification methods, the set is divided into multiple spaces, and the data in each space is non-repetitive. This process is called spatial partition of audit data. After this process, the data space is divided into multiple sub-spaces, and similar division can be made for sub-space, that is multi-scale spatial partition.

**Figure 3:**
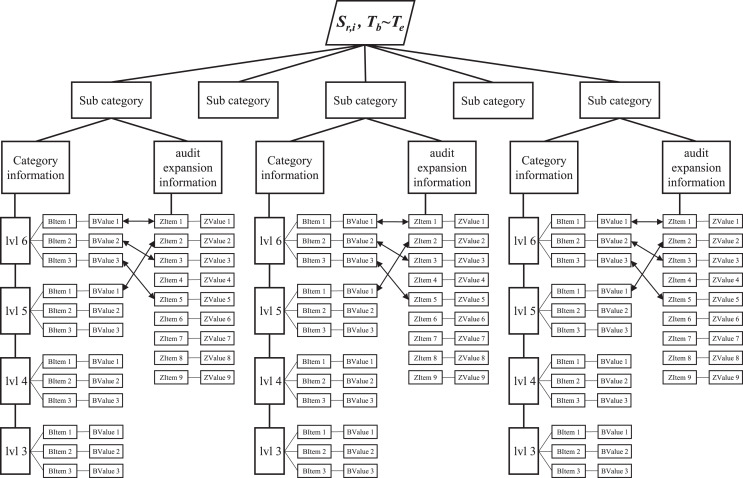
The spatial and temporal structure of audit big data.

At the $r$-level, the audit data can be characterized by three spatio-temporal meta-structures. First, an audit data patch ${S_{r,i}}$ is partitioned at the $r$-level to denote the space, in which the key-value tree exists. Second, the time range ( ${T_b}$ $\sim$ ${T_e}$) is specified to indicate the effective time of the key-value tree, with different scales potentially being applied across different levels and time units. Finally, the key-value tree is organized as a hierarchical structure, as illustrated in Fig. 3.

In this hierarchical structure, the root node is a spatio-temporal fragment of audit data, that consists of ${S_{r,i}}$ and the time range ( ${T_b}$ $\sim$ ${T_e}$), while the intermediate node only denotes the name. The leaf node is structured as key-value pairs. The second layer is classified based on the type of audit data, and the subsequent layer provides detailed information for a specific type of audit data, which can be categorized as either cataloging information or audit expansion information. The cataloging information is divided into 0 to 6 levels according to the categories of audit data, and each level contains multiple key-value pairs of audit data. The audit expansion information is also represented in the form of key-value pairs. The information can be produced through secondary mining based on the cataloging information or generated directly for application.

#### Skeleton-based index

Indexing is a commonly used technique in sorting relevant values in data tables or databases, which expedites the data search process in database. By employing specific index structures, voluminous audit data can be categorized and stored in distinct nodes based on certain categories or layers. This approach ensures data integrity and simplifies dynamic adjustment of audit data for auditors.

**Figure 4:**
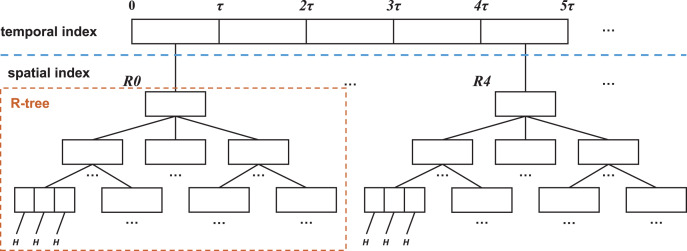
Skeleton index structure.

In audit big data, a *skeleton index* is utilized to retrieve both temporal and spatial information. The index is separated into two parts: the temporal index and the spatial index. The temporal index is achieved using an array or linked list, in which all the elements index the same time period $\tau$. Each element is linked to the root node of an R-tree, which indexes the corresponding spatial data during the time period. The elements in leaf nodes of the R-tree link to the corresponding metadata *H* for the information resource. The structure of the *skeleton index* is illustrated in Fig. 4.

The comprehensive utilization of the skeleton index can simplify the organization structure of audit data, while completely preserving the internal relationship between semantic information and attributes. This approach offers a new method for the efficient storage and management of audit spatio-temporal data, which is characterized by its large volume, rapid change rate, and diverse structure.

#### R-tree-based retrieval

In conventional database systems, HBase (Wiki, 2012) is commonly employed for storage, given its wide application in handling super-large-scale data read-write operations. With its superior reliability, it is capable of maintaining high-performance real-time data retrieval. Nonetheless, HBase falls short of supporting some intricate query conditions.

**Figure 5:**
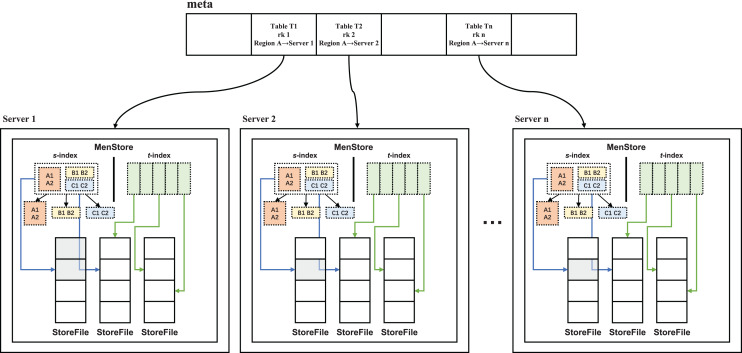
Preliminary sketch of the secondary index structure.

Given the structural characteristics of HBase, a secondary index structure is designed in this study, as depicted in Fig. 5. This structure employs an audit metadata table as the first-level storage (index) and the regional server as the second-level storage (index).

In order to improve the management efficiency of the two-level storage network architecture of HBase, we proposed a two-level storage index method, R-HBase. Specifically, the meta-linked list is used to construct the two-level indexes for the spatial and temporal dimensions, which enables the design of parallel algorithms for both space-time range queries and k-nearest neighbors (kNN) queries (Liu et al., 2019). Range queries are mainly used to retrieve spatiotemporal locations, while kNN queries are used to retrieve specific content in a given spatiotemporal location.

Given a range query $rq$ = ( ${X_\alpha }$, ${Y_\alpha }$, ${X_\beta }$, ${Y_\beta }$, ${T_b}$, ${T_e}$), its purpose is to retrieve all records with a spatial position within ( ${X_\alpha }$, ${Y_\alpha }$, ${X_\beta }$, ${Y_\beta }$) and a temporal value within the range of ( ${T_b}$, ${T_e}$). The main process of the range query involves transforming the spatial predicates ( ${X_\alpha }$, ${Y_\alpha }$, ${X_\beta }$, ${Y_\beta }$) into a set of one-dimensional intervals ${I_{rq}}$ using Hilbert curves (Faloutsos & Roseman, 1989). Subsequently, the corresponding regions are located using the related region server for the region index based on the mapping relationship in the meta-table (HBase, 2012, George, 2011). This article proposes a query optimization method that utilizes the s and t indexes to calculate the selectivity, which aids in filtering out irrelevant data and retaining highly relevant data. Specifically, the spatial predicates are recursively divided using a quadtree, the resulting intervals are intersected with entries in the s index, and the address of the keyword data is calculated.

Algorithm 1 outlines the range query process of R-HBase. In line 1, the spatial predicate is converted to a one-dimensional interval ${I_{rq}}$, while the time predicate is transformed into the interval $[0,T]$ in line 2 to 4 employs the function $findRegions()$ to search for relevant regions intersecting with ${I_{rq}}$. From lines 5 to 8, each corresponding region index is checked to retrieve the result. Line 6 uses R-tree to retrieve data within the query spatial range and returns the addresses of data that meet the conditions. In line 7, the time index is utilized to query data that satisfies the time range and returns a list of data storage addresses. Line 8 queries the intersection of $slist$ and $tlist$ to obtain a list of data storage addresses that meet the time and space requirements. Finally, the data’s detailed information is queried and returned.

**Algorithm 1:** R-HBase range query

**Require:** *rq*= ( ${X_\alpha }$, ${Y_\alpha }$, ${X_\beta }$, ${Y_\beta }$,*T*_*b*_,*T*_*e*_)
**Ensure:** *Qlist* //result list
1: ${I_{rq}} = rectToIntervals({X_\alpha },{Y_\alpha },{X_\beta },{Y_\beta })$
2: $ke{y_s} = {t_s}modT$
3: $ke{y_e} = {t_e}modT$
4: $Regions = findRegions({I_{rq}})$
5: **for** each $region \in Regions$ **do**
6: $slist = region.s\_index.getRecords({X_\alpha },{Y_\alpha },{X_\beta },{Y_\beta })$
7: $tlist = region.tindex.searchIndex({T_b},{T_e})$
8: *Qlist* = intersection(*slit*, *tlist*)
9: **end for**
10: **return** *Qlist*

The kNN query is designed to find a set $R(rq) \subseteq R$ of spatio-temporal data records that satisfy the condition $d(o,({X_{rq}},{Y_{rq}})) \le d(o^\prime,({X_{rq}},{Y_{rq}}))$ for all $o \subseteq R\backslash R(rq)$, where $o.\tau ,o^{\prime}.\tau \in [{T_b},{T_e}]$ and $|R(rq)|$ = $k$. Here, *R* represents a set of spatio-temporal data records, and $rq$ = ( ${X_{rq}}$, ${Y_{rq}}$, ${T_b}$, ${T_e}$, $k$) is the query, where ${X_{rq}}$ and ${Y_{rq}}$ represent the spatio coordinates, ${T_b}$ and ${T_e}$ represent the time range, and $k$ represents the number of nearest neighbors to be searched. The Euclidean distance function is used to calculate the distance between the query point $({X_{rq}},{Y_{rq}})$ and other data points.

To address the issue of high query overhead resulting from the use of n range queries to complete kNN queries, an incremental retrieval method is proposed in this study. The basic workflow of this method involves incrementally retrieving the neighboring points of the target point $({X_{rq}},{Y_{rq}})$ until $k$ results are found (Wang et al., 2010). Specifically, a Hilbert cell $h$ that contains the point $({X_{rq}},{Y_{rq}})$ is first located (Hjaltason & Samet, 1999). Next, all records in $h$ are retrieved using the corresponding regional index, while adjacent cells to $h$ are also retrieved. The records and Hilbert units are then placed in a priority queue, where the priority metric is the distance between the record or Hilbert unit and $({X_{rq}},{Y_{rq}})$. Data with higher priority are continuously queued and processed until they are added to the result list, or the adjacent cells to be queued are retrieved until $k$ results are found.

Algorithm 2 illustrates the process of conducting a KNN query. In line 1, a priority queue *PQ* is initialized, with each element sorted according to the distance from $({X_{rq}},{Y_{rq}})$ to that element. This element can be either a Hilbert unit or a record. If it is a Hilbert unit, its distance is MINDIST. Otherwise, the distance is calculated as the Euclidean distance from $({X_{rq}},{Y_{rq}})$ to the spatial position of the record. Line 2 obtains the Hilbert unit that contains $({X_{rq}},{Y_{rq}})$ and queues it for processing in line 3. Beginning from line 4, the top element $e$ is continuously retrieved from the PO queue (line 5) and processed. When $e$ is a Hilbert unit (line 6), the corresponding region is found from the metadata table (line 7). Then, the relevant region index is searched to retrieve all records that satisfy the time predicate (line 8), and these records are queued into *PQ* (lines 9 to 11). After that, the adjacent unit of $e$ is obtained and queued into *PQ* (lines 12 to 15). If $e$ is a record (line 16), it means $e$ is the result, and $e$ is added to the result list $Qlist$ (line 17). The above process is then repeated until the size of the result list $Qlist$ reaches $k$ (lines 18 to 20).

**Algorithm 2:** R-HBase kNN query

**Require:** *rq* = (*X*_*rq*_,*Y*_*rq*_,*T*_*b*_,*T*_*e*_,*k*)
**Ensure:** *Qlist* //result list
1: $PQ = null$ //initial a priority queue
2: $h = coorToCell({X_{rq}},{Y_{rq}})$
3: $PQ.enqueue(h,MINDIST({X_{rq}},{Y_{rq}}),h)$
4: **while** $PQ \ne \emptyset$ **do**
5: $e = PQ.dequeue()$
6: **if** *e* is typeof cell **then**
7: $rg = findRegions(e)$
8: $RS = rg.findRegions(e,({T_b},{T_e}))$
9: **for** each $record \in RS$ **do**
10: $PQ.enqueue(record,dist(({X_{rq}},{Y_{rq}}),record))$
11: **end for**
12: $CellSet = getNeighborCells(e.center)$
13: **for** each $cell \in CellSet$ **do**
14: $PQ.enqueue(cell,MINDIST(({X_{rq}},{Y_{rq}}),cell))$
15: **end for**
16: **else if***e* is typeof record **then**
17: $Qlist \leftarrow e$
18: **if** $Qlist.size() = k$ **then**
19: **return** *Qlist*
20: **end if**
21: **end if**
22: **end while**

### Chinese to SQL generation model MRo-SQL

Computers are unable to directly comprehend and process natural language, necessitating the development of auxiliary technologies to transform knowledge into a formal model. NL2SQL (Natural Language to SQL) technology is a prevalent conversion tool that enables the transformation of natural language queries into executable SQL statements, enabling users to interact with a database without the need for knowledge of database programming.

Based on the optimization of indexing method and slot filling method described above, we proposed a multi-topic representation learning NL2SQL model, MRo-SQL. The basic framework of this model is a multi-task joint learning architecture, which is designed with an encoder, a column representation module, and a sub-model unit to achieve better generalization performance.

**Figure 6:**
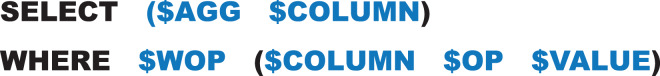
Slot filling method.

The slot-filling method is the basis for NL2SQL generation template, which adheres to the fixed grammar rules of SQL statements, typically structured with SELECT, WHERE, and/or various keywords, followed by corresponding content to be filled. A schematic representation of the slot-filling method is depicted in Fig. 6, in which the black section denotes SQL keywords, and the blue section refers to the content that requires completion.

Although NL2SQL technology can convert natural language into SQL statements, it suffers from several limitations, similar to those encountered in neural networks and other learning approaches, and lacks interpretability of language. It relies on statistical learning, without considering the semantic logic between contexts, making it challenging to adjust and improve. Consequently, traditional NL2SQL technology cannot guarantee the accuracy of SQL statements, which is unacceptable in the audit process. Incorrect SQL statements will delay the audit process. Therefore, to address this issue, we proposed the MRo-SQL model in the system.

**Figure 7:**
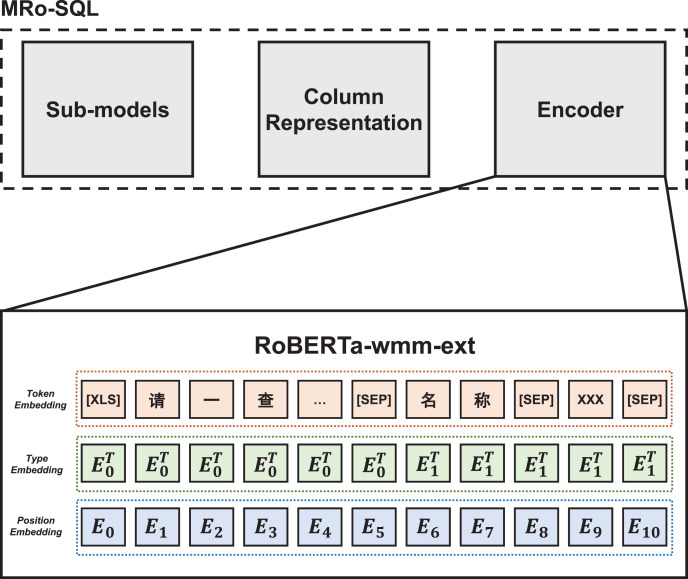
MRo-SQL framework.

The overall structure of the MRo-SQL computing framework is illustrated in Fig. 7. The framework is based on a multi-task joint learning architecture and comprises three main components: the encoder, column representation module, and sub-model unit. To improve the interpretability of the model, the encoder utilizes the RoBERTa-wwm-ext model, which is trained on a larger *corpus* compared to the general Bert and has better generalization performance. The proposed architecture addresses the bottleneck of NL2SQL technology, which is database field judgment, by incorporating a context enhancement layer that serves as the semantic representation of database columns. However, the limitations of NL2SQL technology, such as the lack of interpretability, remain unresolved, and the proposed MRo-SQL model is designed to address this issue.

The column representation module is designed to enhance the semantic representation of table headers. The input sequence is encoded by RoBERTa, which generates a semantic encoding vector. Since there is only one sequence of query statements per input, there may be gaps between multiple sequences of database table fields. To address this issue, MRo-SQL leverages global information and applies attention mechanism to the table headers, which results in improved semantic representation.

In the sub-model unit of the MRo-SQL model, RoBERTa semantic encoding and database column information expression are employed to make predictions. Consistent with prior research, NL2SQL tasks are decomposed into multiple sub-models. However, the MRo-SQL model decomposes the task into six subtasks, which are based on data from WikiSQL.

The MRo-SQL model employs a multitask joint training framework to simultaneously train multiple subtasks. In real-world scenarios, natural language queries from users may not explicitly contain the target VALUE value, but may instead only include synonyms of the target VALUE value within the “string type” field. For such cases, the extracted VALUE value from the natural language queries cannot be directly applied to fill the SQL template. To address this issue, we employed a rule-matching method for post-processing the VALUE values. Specifically, we selected the content with the highest similarity to VALUE from the database as the filling VALUE, using the Route-L Rouge (Recall-Oriented Understudy for Gisting Evaluation) method for similarity calculation. The Rouge method comprises a group of string similarity calculation techniques that were first used in research on automatic summarization, and evaluates the quality of summary generation based on the similarity between automatically generated summaries and reference summaries.

## Results

In this section, we conduct an evaluation of the index construction and retrieval optimization performance, and verify the effectiveness of the MRo-SQL model.

### Experimental setup

The experiment mainly included the evaluation of the index building and retrieval optimization performance, as well as the effectiveness of the MRo-SQL model.

To evaluate the performance of index construction and retrieval optimization, we used MD-HBase (Nishimura et al., 2011) with functions similar to those of the baseline method. Two parameters were introduced to test the algorithm under various conditions. The first was the selectivity $\Phi$, which is defined as:

$\Phi = {{Le{n_{\left( {{T_b},{T_e}} \right)}}} \over {Le{n_\tau }}} \cdot {{Ar{e_{rq}}} \over {Ar{e_s}}}$

where, $Le{n_{({T_b},{T_e})}}$ represents the length of the query time range $({T_b},{T_e})$. $Le{n_\tau }$ represents the length of the time range. $Ar{e_{rq}}$ represents the area of the query space range $rq$ and $Ar{e_s}$ represents the area of the entire space. The size of the query scope is specified. When $\Phi$ is large, the spatio-temporal is more involved.

The other parameter was the cluster size, which is defined by the number of RegionServers in the cluster. In HBase, data is partitioned into multiple regions, and each region is managed by a specific RegionServer. If the number of RegionServers is too small, the cluster may experience uneven data distribution, leading to potential performance bottlenecks. Therefore, to enhance the system’s performance and scalability, increasing the number of RegionServers in the HBase cluster is an effective way to achieve load balancing and improve concurrent processing capabilities.

In this experiment, we initially set the values of $\Phi$ and cluster size as 10% and 9, respectively. A total of 10 queries with different temporal and spatial ranges were performed for each value of $\Phi$ and cluster size, and the average response time was used as a performance metric.

To assess the performance of the MRo-SQL model, we utilized TableQA and WikiSQL (Zhong, Xiong & Socher, 2017), which are currently the largest public datasets for single-table SQL parsing in both English and Chinese. The TableQA dataset contains financial and general field data for single-table SQL parsing scenarios, and the SQL statements corresponding to natural language queries were constructed through manual annotation. It is currently the largest Chinese dataset for SQL parsing and includes 54,059 manually annotated samples.

WikiSQL is the largest SQL parsing dataset at present. It is oriented to a single-table SQL parsing scenario and contains 24,241 data tables, 80,654 manually labeled natural-language questions, and SQL-statement pairs from Wikipedia.

Accuracy was used as a metric to evaluate the prediction results. It was calculated as follows:

$\eqalign{
  & score = \left\{ {\matrix{
   {0,{y^\prime } \ne y}  \cr 
   {1,{y^\prime } = y}  \cr 

 } } \right.  \cr 
  & acc = {1 \over N}\sum\limits_{i = 1}^N s cor{e_i} \cr} $

where, *N* represents the number of samples, $y^{\prime}$ represents the predicted value, $y$ represents the true value, $score$ represents the sample score, and $acc$ represents the accuracy of the data evaluation.

SQLNet (Xu, Liu & Song, 2017), Coarse2Fine (Dong & Lapata, 2018), SQLova (Hwang et al., 2019) and X-SQL (He et al., 2019) were used as baseline comparison models in the experiment. SQLNet is a neural network-based model for generating SQL queries directly from natural language questions. It uses a multi-task learning approach to jointly predict query components and types, and has achieved state-of-the-art performance on the WikiSQL dataset. Coarse2Fine is a two-stage neural network model for generating SQL queries. The first stage generates a coarse sketch of the query, and the second stage refines the sketch to produce the final query. This approach has been shown to improve the accuracy of SQL query generation on the Spider dataset. SQLova is a sequence-to-sequence model that generates SQL queries by directly predicting the SQL tokens from natural language questions. It uses a copy mechanism to handle out-of-vocabulary tokens and a two-stage training process to improve the model’s accuracy. SQLova has achieved state-of-the-art performance on the Spider dataset. X-SQL is a cross-lingual neural network-based model for generating SQL queries from natural language questions in multiple languages. It uses a shared encoder to learn a language-independent representation of the input question and a language-specific decoder to generate the SQL query. X-SQL has been shown to achieve high accuracy on cross-lingual SQL query generation tasks. These four baseline models achieved good performance on the WikiSQL dataset.

### Index construction and retrieval optimization

As R-HBase was derived from MD-HBase, we selected MD-HBase and STEHIX, a model with comparable capabilities, as our benchmark methods. These benchmark models have demonstrated outstanding results in previous research experiments.

#### Range query

**Figure 8:**
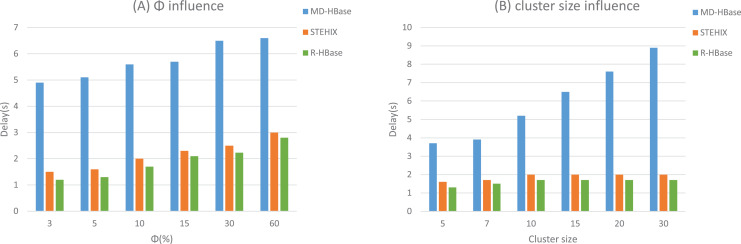
Range query experimental results.

For the range query, we gradually increased $\Phi$ from 3% to 50% as illustrated in Fig. 8A. The response time of the three models, STEHIX, MD-HBase, and R-HBase, all increased in proportion to the increase of $\Phi$. This can be attributed to the selective definition of the query range size; the larger the value of $\Phi$, the more space-time records it encompasses, leading to an increase in index processing time. The experimental results indicate that the R-HBase model outperforms the other two models in terms of response time, which can be attributed to the design of its regional index. This further confirms the suitability of R-Tree for spatial retrieval. Although MD-HBase implements an index in the metadata, it does not establish an index for the internal structure of the region. As a result, scanning for results consumes a significant amount of resources. Our R-HBase model is optimized for HBase’s two-level architecture and can effectively search each region using spatial indexing, resulting in a significant improvement in performance.

As shown in Fig. 8B, a comparison experiment was conducted to evaluate the response time of R-HBase and other algorithms across various cluster sizes, which ranged from five to 30. The results indicate that R-HBase and STEHIX have comparable response times and excellent scalability, performing impressively. As the cluster size expands, additional RegionServers are involved in the processing and collaborate using their regional indices in parallel. Nevertheless, the scalability of MD-HBase is hindered by the absence of the index StoreFiles feature, leading to a noticeable increase in response time as the cluster size grows.

#### kNN query

**Figure 9:**
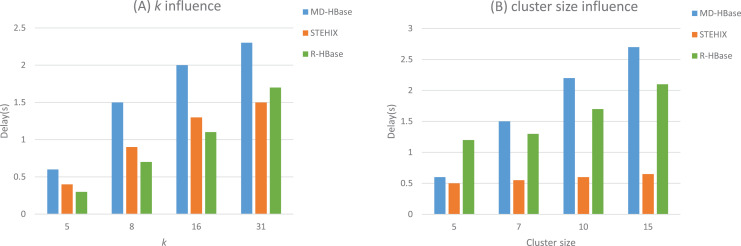
kNN query experimental results.

For the kNN query, the default values for $k$ and cluster size are eight and nine, respectively. We increased the value of $k$ from 4 to 31. The results in Fig. 9A shows that R-HBase outperforms STEHIX and MD-HBase. As $k$ increases, both R-HBase and the comparison algorithms require more time to process the query. R-HBase takes less time to retrieve $k$ results, which can be explained by the positive role played by the R-Tree spatial index embedded within the R-HBase region.

As shown in Fig. 9B, the cluster size ranges from five to 30. The results of the experiments indicate that R-HBase continues to surpass STEHIX and MD-HBase. Just like the results obtained in the range query experiments, R-HBase and STEHIX display similar response times and excellent scalability, exhibiting strong performance, while the response time of MD-HBase displays a noticeable increase as the cluster size increases.

#### Cluster distribution comparison

In the R-HBase framework, we have introduced R-Tree as the spatial index module, which is based on HBase storage. This design allows R-HBase to effectively tackle the data hotspot problem of Hadoop distributed cluster nodes. The audit metadata table serves as the first-level storage (index), while the region server functions as the second-level storage (index). The spatial encoding of the audit time-space structure is used as the content of the metadata table (meta table). The region server stores the specific content of the data time-space structure and relies on the MemStore structure storage as the audit data time-space index, which directly points to the storage file of the audit data storage time-space structure.

**Figure 10:**
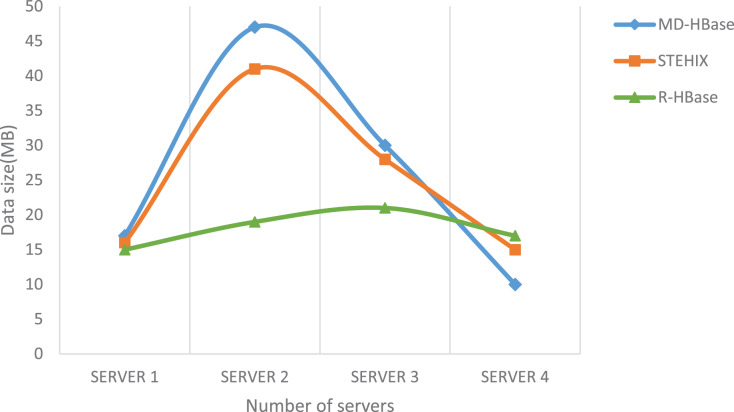
Data size of different cluster servers.

Figure 10 illustrates the distribution of 100 GB of randomly selected data from the simulated dataset across various nodes. This data was stored on nodes within the DataNode cluster using the MD-HBase, STEHIX, and R-HBase frameworks. The results of the comparison experiment indicate that the data distribution in R-HBase exhibits a relatively uniform distribution across nodes, whereas the distribution of data in the STEHIX and MD-HBase index frameworks is less balanced.

The reason why the data distribution in R-HBase appears to be relatively balanced is explained by the spatial part allocation algorithm in the R-HBase index framework. For the spatial component of the data stored in R-HBase region servers, which house the time-space metadata based on HBase, we employ R-Tree to enhance storage efficiency. If a method of dividing the space into equal-sized rectangular regions was utilized to create the spatial index, the data distribution in the Hadoop cluster nodes would not be uniform. This is because trajectory objects only move within sections, and there is a significant variation in the amount of data between sections, leading to data imbalance issues among different cluster nodes.

However, in the R-HBase index framework, R-Tree is used to improve storage performance. R-Tree encloses spatial objects within the smallest rectangle parallel to the coordinate axis and creates a spatial index through the minimum bounding rectangle. The virtual bounding rectangle directory design encapsulates the spatial objects within the spatial rectangle and serves as the spatial index, including pointers to the encompassed spatial entities. The R-Tree spatial index is based on the rectangle that contains the entities, and the hierarchy of R-Tree conveys the resolution information. When executing a query task, finding the smallest rectangle that contains the spatial object is sufficient, effectively avoiding any potential data hotspot issues.

### MRo-SQL model validity

In this section, we evaluated the performance of MRo-SQL model. TableQA and WikiSQL were used as SQL parsing experimental datasets.

Tables 1 and 2 present the experimental results of the SQLNet, Coarse2Fine, SQLove, X-SQL, and MRo-SQL models for the TableQA and WikiSQL datasets, respectively. Here, $ac{c_{LF}}$ represents the logical-form accuracy, $ac{c_{EX}}$ represents the execution accuracy, and $ac{c_{MEAN}}$ represents the mean accuracy. The experimental results indicated that the proposed MRo-SQL model achieved the best performance for the TableQA and WikiSQL datasets. For the TableQA test data, MRo-SQL performed matching with exact accuracy.

**Table 1:** Parsing representation of MRo-SQL and baseline model on TableQA.

Model	Verification set (%)			Test set (%)		
	$ac{c_{LF}}$	$ac{c_{EX}}$	$ac{c_{MEAN}}$	$ac{c_{LF}}$	$ac{c_{EX}}$	$ac{c_{MEAN}}$
SQLNet	60.1	64.9	62.5	61.3	66.0	64.7
Coarse2Fine	71.5	75.4	74.5	72.7	76.4	74.6
SQLova	79.8	84.6	81.7	81.1	84.7	82.9
X-SQL	81.2	85.3	84.3	82.4	86.4	84.5
MRo-SQL	87.6	91.0	89.2	89.1	92.2	92.6

**Table 2:** SQL parsing representation of MRo-SQL and baseline model on WikiSQL.

Model	Verification set (%)			Test set (%)		
	$ac{c_{LF}}$	$ac{c_{EX}}$	$ac{c_{MEAN}}$	$ac{c_{LF}}$	$ac{c_{EX}}$	$ac{c_{MEAN}}$
SQLNet	–	65.7	–	–	64.0	–
Coarse2Fine	68.2	74.4	72.3	67.5	73.9	71.6
SQLova	76.8	82.1	79.4	76.0	81.1	78.6
X-SQL	78.9	84.2	81.6	78.4	83.5	80.9
MRo-SQL	84.5	90.4	87.3	84.5	90.1	87.2

The SQLNet model employs sequence-to-set technology and represents the first template-filling model applied to the WikiSQL dataset. It aims to solve the issue of sensitivity to the order of SQL components in previous parsing schemes and lays the foundation for subsequent research. In a similar vein, the proposed MRo-SQL model is also a template-filling model that outperforms SQLNet in terms of $ac{c_{EX}}$, with a 40.2% improvement for TableQA and a 37.6% improvement for WikiSQL test data. Coarse2Fine is a sequence generation model based on sequence-to-sequence technology. Compared with Coarse2Fine, MRo-SQL shows a significant increase of 20.7% and 21.5% in $ac{c_{EX}}$ for the TableQA and WikiSQL test data, respectively. SQLova utilizes pre-trained BERT as an encoder and represents an upgraded version of the SQLNet model. Experimental results comparing SQLova and SQLNet demonstrate the significant improvement in SQL parsing achieved by pre-training.

The structure of the MRo-SQL model bears resemblance to that of X-SQL, albeit with a notable difference in the VALUE extraction subtask design. While X-SQL extracts the VALUE associated with the field based on the field information, MRo-SQL extracts all the VALUE directly and subsequently determines the association between the VALUE and the field. In the TableQA test set, MRo-SQL achieved a 5.8% improvement in $ac{c_{MEAN}}$ compared to X-SQL, indicating its superior ability to handle multiple VALUE extraction problems. On the WikiSQL dataset, the MRo-SQL model performed even better, achieving a $ac{c_{MEAN}}$ improvement of 7.0% and 7.8% for the verification set and test set, respectively. This finding suggests that the MRo-SQL model can deliver better performance for SQL parsing tasks even without multiple VALUE extraction problems. Moreover, compared to X-SQL, MRo-SQL exhibited stronger robustness.

MRo-SQL and X-SQL are both template-filling models. To further evaluate the performance of the models, we compared and analyzed the performance of MRo-SQL and X-SQL on multiple subtasks.

In the WikiSQL dataset, the SELECT component was restricted to only one sub-fragment. Moreover, the conditional relationship in the WHERE component was limited to “AND”, and the W-NUM-OP subtask was only required to predict the number of sub-fragments in the WHERE component without predicting the conditional relationship. The W-VALUE-MATCH subtask was formed by combining the W-VALUE and W-MATCH subtasks. During the test phase, there existed an order dependency between the subtasks when generating SQL statements. For instance, the upstream task of S-COL-AGG was S-COL, where the model first predicted the selected columns in the SELECT component through the S-COL subtask, and then it predicted the associated aggregation operation of the selected columns *via* the S-COL-AGG subtask. If the prediction of the upstream task was incorrect, the downstream task’s prediction would become meaningless. Therefore, the performance of the downstream task was evaluated using conditional probability. Specifically, for S-COL-AGG, its subtask’s performance was calculated as follows:

$p = {{{n_2}} \over {{n_1}}}$

where ${n_1}$ represents that the S-COL subtask in the test set predicts the correct sample number, and ${n_2}$ represents the correct sample number that the S-COL subtask and the S-COL-AGG subtask in the test set predict simultaneously. There are three pairs of sequential dependent subtasks: S-COL, S-COL-AGG, W-COL, W-COL-OP, W-COL, and W-VALUE-MATCH.

As presented by Table 3, MRo-SQL achieved the best performance in many subtasks. Compared with X-SQL, MRo-SQL achieved accuracy improvements of 2.75% and 5.97% for the W-COL and W-VALUE-MATCH subtasks, respectively, for TableQA dataset. The experimental results for the W-VALUE-MATCH subtask showed that the VALUE extraction method in the MRo-SQL model can effectively deal with multiple VALUE extraction problems and compensate for the shortcomings of previous VALUE extraction methods based on field information. For the WikiSQL dataset, the MRo-SQL and X-SQL models performed poorly on the S-COL-AGG subtasks, achieving only 90% accuracy. Considering the performance of the W-COL-OP subtask for the two datasets and the S-COL-AGG subtask for TableQA, we concluded that the poor performance of the S-COL-AGG subtask for the WikiSQL dataset was mainly caused by dataset annotation; additionally, the WikiSQL dataset contained numerous data with chaotic annotations of aggregation operations.

**Table 3:** SQL parsing representation of MRo-SQL and baseline model on WikiSQL.

Sub-task	TableQA (%)		WikiSQL (%)	
	X-SQL	MRo-SQL	X-SQL	MRo-SQL
S-NUM	97.52	99.71	–	–
S-COL	96.29	98.50	95.48	97.49
S-COL-AGG	97.19	99.33	88.71	90.55
W-NUM-OP	95.28	97.23	96.70	98.71
W-COL	95.08	97.69	94.23	96.44
W-COL-OP	97.67	99.64	97.14	99.18
W-VALUE-MATCH	90.73	96.15	97.31	99.56
$ac{c_{EX}}$	86.42	91.54	83.54	89.50

## Conclusion and future work

This article presents BDMCA, a big data management system for Chinese audits. A mediator-wrapper based hybrid data management framework is designed, to manage heterogeneous audit data uniformly. Besides, a skeleton index and an R-HBase spatio-temporal meta-structure are proposed, to efficiently link spatio-temporal indexes and indexes of diverse information types. Morevoer, a knowledge-based Chinese-assisted auditing model, MRo-SQL, is developed, which optimizes the NL2SQL technology generation process and improves the accuracy of generating SQL statements. We evaluated BDMCA on public available dataset, and got satisfying performance.

This study investigated the management and service technologies for knowledge-driven Chinese audit big data, including audit knowledge modeling, data standardization and unification, data storage and retrieval, and query language conversion. Our research has a broad scope, and the various specific technologies require further investigation. Firstly, Chinese big data management needs to be improved. The retrieval efficiency can be enhanced by load balancing, which will allow for the development of better Chinese-aided audit model generation technology and improve the model’s accuracy. Secondly, we examined the potential applications of audit intelligence. With the growing popularity of big data technology in China, various machine learning and deep learning methods can be applied to audits. For example, article documents can be digitized *via* text recognition, and data mining technology can be used to uncover the hidden information behind big data and identify audit clues.

## Supplemental Information

10.7717/peerj-cs.1317/supp-1 Supplemental Information 1 Two-level storage structure HBase code.

10.7717/peerj-cs.1317/supp-2 Supplemental Information 2 R-tree indexing.

## Additional Information and Declarations

### Data Availability

The following information was supplied regarding data availability: The code are available in the Supplemental Files. The dataset is available at the US Census Bureau: https://www.census.gov/programs-surveys/geography/guidance/tiger-data-products-guide.html.

## References

[ref-1] Abouzeid A, Bajda-Pawlikowski K, Abadi D, Silberschatz A, Rasin A (2009). Hadoopdb: an architectural hybrid of mapreduce and dbms technologies for analytical workloads. Proceedings of the VLDB Endowment.

[ref-2] Devlin J, Chang MW, Lee K, Toutanova K (2018). Bert: pre-training of deep bidirectional transformers for language understanding. ArXiv preprint.

[ref-3] Dong L, Lapata M (2018). Coarse-to-fine decoding for neural semantic parsing. ArXiv preprint.

[ref-4] Doulkeridis C, Vlachou A, Kotidis Y, Vazirgiannis M (2007). Peer-to-peer similarity search in metric spaces.

[ref-5] Faloutsos C, Roseman S (1989). Fractals for secondary key retrieval.

[ref-6] Fan X, Monti E, Mathias L, Dreyer M (2017). Transfer learning for neural semantic parsing. ArXiv preprint.

[ref-7] George L (2011). HBase: the definitive guide: random access to your planet-size data.

[ref-35] Green BF, Wolf AK, Chomsky C, Laughery K (1961). Baseball: an automatic question-answerer.

[ref-8] Guo T, Gao H (2019). Content enhanced bert-based text-to-sql generation. ArXiv preprint.

[ref-9] HBase A (2012). Apache hbase reference guide. https://hbase.apache.org/book.html.

[ref-10] He P, Mao Y, Chakrabarti K, Chen W (2019). X-sql: reinforce schema representation with context. ArXiv preprint.

[ref-11] Hernandez C, Rodriguez MA, Marin M (2008). A p2p meta-index for spatio-temporal moving object databases.

[ref-12] Hjaltason GR, Samet H (1999). Distance browsing in spatial databases. ACM Transactions on Database Systems (TODS).

[ref-13] Holt T, Loraas TM (2021). A potential unintended consequence of big data: does information structure lead to suboptimal auditor judgment and decision-making? Does information structure lead to suboptimal auditor judgment.

[ref-14] Hwang W, Yim J, Park S, Seo M (2019). A comprehensive exploration on wikisql with table-aware word contextualization. ArXiv preprint.

[ref-15] Jia R, Liang P (2016). Data recombination for neural semantic parsing. ArXiv preprint.

[ref-16] Liang P, Jordan MI, Klein D (2013). Learning dependency-based compositional semantics. Computational Linguistics.

[ref-17] Liu L, Su J, Liu X, Chen R, Huang K, Deng RH, Wang X (2019). Toward highly secure yet efficient knn classification scheme on outsourced cloud data. IEEE Internet of Things Journal.

[ref-18] Nah Y, Lee J, Lee WJ, Lee H, Kim MH, Han KJ (2005). Distributed scalable location data management system based on the galis architecture.

[ref-19] Nishimura S, Das S, Agrawal D, El Abbadi A (2011). Md-hbase: a scalable multi-dimensional data infrastructure for location aware services.

[ref-20] Özsu MT, Valduriez P (1999). Principles of Distributed Database Systems.

[ref-21] Quezada-Sarmiento PA, Alvarado-Camacho PE, Chango-Cañaveral PM (2017). Development of an information system audit in a data center: implementation of web application to the management of audited elements.

[ref-22] Radford A, Narasimhan K, Salimans T, Sutskever I (2018). Improving language understanding by generative pre-training. https://cdn.openai.com/research-covers/language-unsupervised/language_understanding_paper.pdf.

[ref-23] Sarzynska-Wawer J, Wawer A, Pawlak A, Szymanowska J, Stefaniak I, Jarkiewicz M, Okruszek L (2021). Detecting formal thought disorder by deep contextualized word representations. Psychiatry Research.

[ref-24] Tian Y, Zou T, Ozcan F, Goncalves R, Pirahesh H (2015). Joins for hybrid warehouses: exploiting massive parallelism in hadoop and enterprise data warehouses.

[ref-25] Tomasic A, Raschid L, Valduriez P (1998). Scaling access to heterogeneous data sources with disco. IEEE Transactions on Knowledge and Data Engineering.

[ref-26] Trajcevski G, Ding H, Scheuermann P, Cruz IF (2007). Bora: routing and aggregation for distributed processing of spatio-temporal range queries.

[ref-27] Wang J, Wu S, Gao H, Li J, Ooi BC (2010). Indexing multi-dimensional data in a cloud system.

[ref-28] Wiki H (2012). Hbase: bigtable-like structured storage for hadoop hdfs. http://wiki.apache.org/hadoop/Hbase.

[ref-29] Xu Y, Kostamaa P, Gao L (2010). Integrating hadoop and parallel dbms.

[ref-30] Xu X, Liu C, Song D (2017). Sqlnet: generating structured queries from natural language without reinforcement learning. ArXiv preprint.

[ref-31] Yoon K, Hoogduin L, Zhang L (2015). Big data as complementary audit evidence. Accounting Horizons.

[ref-32] Zeyu H, Geming X, Zhaohang W, Sen Y (2020). Survey on edge computing security.

[ref-33] Zhong V, Xiong C, Socher R (2017). Seq2sql: generating structured queries from natural language using reinforcement learning. ArXiv preprint.

